# 5H-Benzo[d]Benzo[4,5]Imidazo[2,1-b][1,3]Thiazine as a Novel Electron-Acceptor Cored High Triplet Energy Bipolar Host Material for Efficient Solution-Processable Thermally Activated Delayed Fluorescence Organic Light-Emitting Diodes

**DOI:** 10.3389/fchem.2020.00061

**Published:** 2020-02-07

**Authors:** Mallesham Godumala, Jiwon Yoon, Seo Yeon Park, Chiho Lee, Youngseo Kim, Ji-Eun Jeong, Sungnam Park, Han Young Woo, Min Ju Cho, Dong Hoon Choi

**Affiliations:** Department of Chemistry, Research Institute for Natural Sciences, Korea University, Seoul, South Korea

**Keywords:** new electron-acceptor core, bipolar hosts, thermally activated delayed fluorescence, solution process, organic light emitting diodes

## Abstract

Organic entities that can transport electrons are seldom available to develop adequate bipolar host materials applicable for solution-processable thermally activated delayed fluorescence (TADF)-organic light-emitting diodes (OLEDs). Therefore, the introduction of new electron-affine entities that plausibly demonstrate high triplet energy (*E*_T_) is of urgent need. In this contribution, we introduced benzimidazo[1,2-a][3,1]benzothiazine (BBIT) as a novel electron-affine entity and developed two new bipolar host materials, CzBBIT and 2CzBBIT. Both host materials exhibit high *E*_T_ of 3.0 eV, superior thermal robustness with the thermal decomposition temperature of up to 392°C, a glass transition temperature of up to 161°C, and high solubility in common organic solvents. Consequently, the solution-processable OLEDs fabricated using a recognized IAcTr-out as the green TADF emitter doped into CzBBIT as the host, realized a maximum external quantum efficiency (EQE) of 23.3%, while the 2CzBBIT:IAcTr-out blend film-based device displayed an EQE of 18.7%. These outcomes corroborated that this work could shed light on the scientific community on the design of new electron-affine entities to establish the effective use of bipolar host materials toward proficient solution-processable TADF-OLEDs.

**Graphical Abstract:**
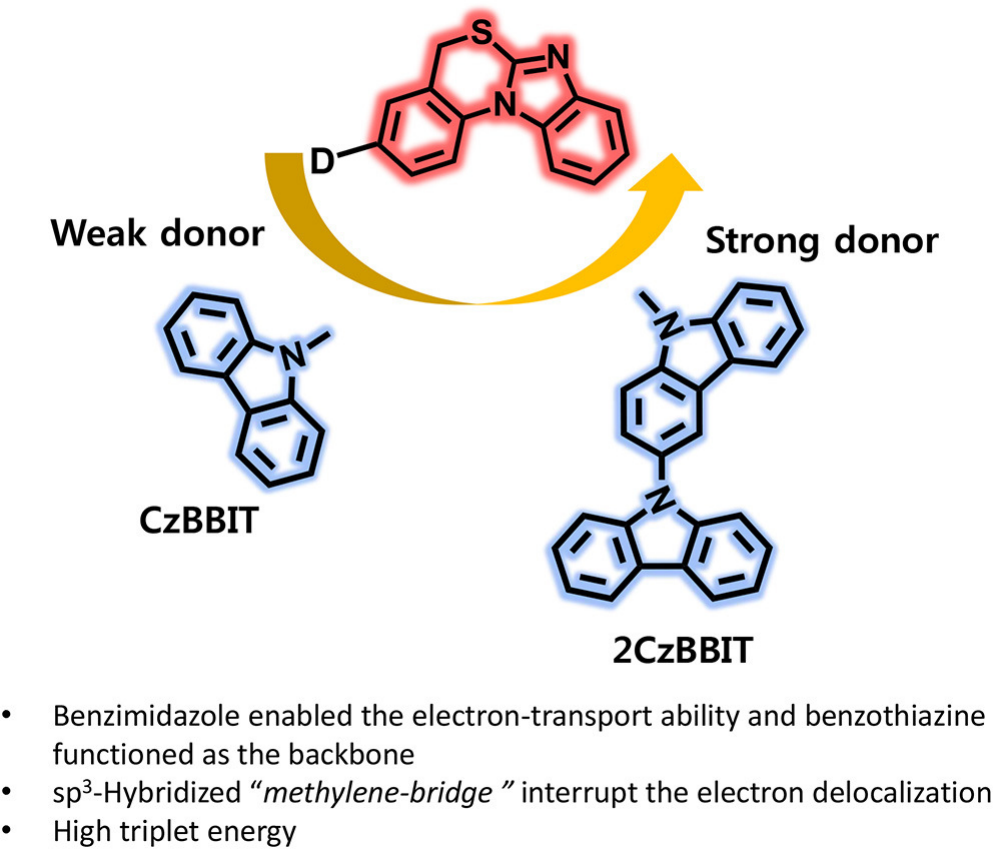
Benzimidazo[1,2-a][3,1]benzothiazine introducing as an electron acceptor for the first time and developed two new bipolar host materials (CzBBIT and 2CzBBIT) for solution-processable thermally activated delayed fluorescence OLEDs.

## Introduction

Metal-free organic molecules that exhibit thermally activated delayed fluorescence (TADF) properties have attracted a great deal of attention with their breakthrough performances in organic light-emitting diodes (OLEDs) (Uoyama et al., 2012; Godumala et al., 2016; Ahn et al., 2019; Kondo et al., 2019). The TADF materials render the potential to utilize both singlet and triplet excitons through reverse intersystem crossing (RISC) by virtue of the minimal energy gap between the singlet and triplet excited states (Δ*E*_ST_), resulting in a theoretical internal quantum efficiency approaching 100% (Uoyama et al., 2012). In contrast, phosphorescent OLEDs (PhOLEDs) exhibit excellent device performance, but there are still improvements to be made such as the use of rare earth metal complexes, their toxicity, and high manufacturing costs. Consequently, TADF materials are the best alternatives to PhOLEDs in terms of efficiency and cost. Similar to phosphorescent emitters, TADF emitters also suffer from concentration quenching through triplet-triplet annihilations and triplet-polaron annihilations owing to the long lifetime of triplet excitons (τ_d_), which could be suppressed by uniformly dispersing into a suitable host matrix (Godumala et al., 2018, 2019; Chatterjee and Wong, 2019). Therefore, both hosts and emitters contribute equally toward the high performance of OLEDs. However, the host materials have not been explored much compared to the emitters.

In recent years, various TADF materials processed through vacuum deposition have been developed, and the majority of them have already been employed to achieve high external quantum efficiency (EQE) of over 25.0% (up to 38.0%) (Uoyama et al., 2012; Ahn et al., 2019; Kondo et al., 2019). In spite of the high performances of vacuum processed devices, the OLEDs fabricated through the solution process have many unique advantages, including an easy fabrication process, mass production of large-scale products, easy control of dopant concentration and low-cost manufacturing (Shao et al., 2017; Huang et al., 2018; Zou et al., 2018; Tsai et al., 2019).

Several host materials reported in the literature perform a monopolar-type (predominant hole-transport) behavior (for instance, CBP, mCP, mCBP, and SiCz) due to the lack of sufficient electron-transport entities. The monopolar behavior of hosts can move the charge recombination zone close to the adjacent transport layers, which can be detrimental to the device performance as well as color purity. Therefore, monopolar behavior and poor thermal stability of these host materials restrict their widespread applications in OLEDs (Godumala et al., 2018). In contrast, host materials constructed of the donor-acceptor structure can enable balanced charge-carrier transport, which can broaden the exciton recombination zone in the emissive layer, thereby boosting the overall device performance (Cui et al., 2016; Kim et al., 2017; Jung et al., 2019; Konidena et al., 2019). Thus, far, numerous host materials reported in TADF-OLEDs have been designed by molecular engineering or by changing the ratio or position of the known donor and acceptor entities (Fan et al., 2015; Zhang et al., 2016a,b; Jeon et al., 2018). Very few new electron-acceptor cores are reported to develop bipolar host materials in OLEDs. For instance, Adachi et al. demonstrated a new rigid electron-deficient benzimidazobenzothiazole (BID-BT) and developed two new bipolar host materials, 29Cz-BID-BT and 39Cz-BID-BT (Cui et al., 2016). By employing them as hosts and a familiar DPAC-TRZ as a blue TADF emitter, the devices achieved an EQE of approximately 21.0%. Later, our group introduced chromenopyrazole (CP) as the new electron-acceptor entity and developed two new bipolar hosts by appending carbazole or bicarbazole as donors (CzCP and 2CzCP). The devices employing CzCP as the host, and the well-known TCzTrz as the blue TADF emitter realized a maximum EQE of 27.9% [CIE coordinates of (0.15, 0.21)] (Godumala et al., 2018). However, the aforementioned host materials were deposited through vacuum thermal evaporation owing to their poor solubility. Therefore, the exploration of new electron-acceptor cores for application as suitable bipolar host materials that demonstrate high solubility is imperative in promoting solution-processable OLEDs.

In this study, we introduced another new electron-deficient heterocyclic entity, benzimidazo[1,2-a][3,1]benzothiazine (BBIT), for the first time and developed two new solution-processable bipolar host materials, namely 3-(9H-carbazol-9-yl)-5H-benzo[d]benzo[4,5]imidazo[2,1-b][1,3]thiazine (CzBBIT) and 3-(9H-[3,9′-bicarbazol]-9-yl)-5H-benzo[d]benzo[4,5]imidazo[2,1-b][1,3]thiazine (2CzBBIT), which were integrated with electron-rich carbazole or bicarbazole entities. The electron-deficient benzimidazole enabled the electron-transport property, while benzothiazine functioned as the backbone to BBIT. The existence of a sp^3^-hybridized methylene bridge could interrupt the electron delocalization between the electron donor and acceptor cores to some extent, and was, therefore, advantageous to retain high triplet energy (*E*_T_) and partially enables solubility. The synthesis of BBIT is moderately facile and rather economical, because expensive catalysts, such as palladium and gold, are not employed. As already envisaged from the design principles, both these compounds manifested a high *E*_T_ (~3.0 eV), adequate highest occupied molecular orbital (HOMO) and lowest unoccupied molecular orbital (LUMO) energies, high solubility, and an excellent thermal stability and homogeneous film formation, which enabled their application as host materials for OLEDs. Consequently, the solution-processable OLEDs were fabricated by implementing these new materials as hosts and IAcTr-out as the green TADF emitter (Park et al., 2018). The CzBBIT and 2CzBBIT accomplished a remarkably high device performance with EQE values as high as 23.3 and 18.7%, respectively. This first report demonstrating a breakthrough performance corroborates that BBIT is an outstanding electron-acceptor core to develop versatile host materials for solution-processable OLEDs in the near future.

## Experimental

### Synthesis

#### Synthesis of 3-(9H-Carbazol-9-yl)-5H-Benzo[d]Benzo[4,5]Imidazo[2,1-b][1,3]Thiazine (CzBBIT)

1*H*-benzo[d]imidazole-2-thiol (0.5 g, 3.3 mmol), CuI (63 mg, 0.33 mmol), and Cs_2_CO_3_ (2.17 g, 6.7 mmol) were mixed in dimethylformamide (DMF, 20 mL) and stirred for 20 min under nitrogen atmosphere. Compound 4a (1.68 g, 3.3 mmol) in DMF (20 mL) and L-proline (77 mg, 0.67 mmol) were subsequently added and stirred at 110°C for 8 h. The mixture was poured into deionized water after cooled down to room temperature (RT), and extracted with ethyl acetate. The organic extracts were dried over Na_2_SO_4_ and concentrated *in vacuo*, and the residue was chromatographed by eluting with methylene chloride (MC) to afford CzBBIT as a white solid. The compound was further purified by precipitating in MC: hexane (1:3) (1.2 g, 89% yield). ^1^H NMR (500 MHz, CDCl_3_): δ (ppm) 8.18-8.14 (m, 2H), 8.07 (d, *J* = 8.5 Hz, 1H), 7.91-7.86 (m, 1H), 7.82-7.77 (m, 1H), 7.68 (dd, *J* = 8.5, 2.1 Hz, 1H), 7.61 (d, *J* = 2.1 Hz, 1H), 7.47-7.41 (m, 4H), 7.39-7.35 (m, 2H), 7.34-7.29 (m, 2H), 4.10 (s, 2H). ^13^C NMR (125 MHz, CDCl_3_): δ (ppm) 150.40, 144.02, 140.73, 135.18, 134.39, 132.61, 127.52, 127.48, 126.61, 126.17, 123.76, 123.52, 123.33, 120.49, 120.38, 119.77, 119.45, 111.36, 109.55, 30.32. MS (MALDI-TOF) [m/z]: Calcd for C_26_H_17_N_3_S, 403.114; Found, 403.115 [M^+^]. Elemental analysis (%) calcd for C_26_H_17_N_3_S: C 77.39, H 4.25, N 10.41, S 6.60. Found: C 77.26, H 4.38, N 10.30, S 6.73.

#### Synthesis of 3-(9H-[3,9′-bicarbazol]-9-yl)-5H-benzo[d]benzo[4,5]imidazo[2,1-b][1,3]thiazine (2CzBBIT)

The synthetic procedure described for CzBBIT was adopted to afford 2CzBBIT by combining 1*H*-benzo[d]imidazole-2-thiol (0.5 g, 3.3 mmol), CuI (63 mg, 0.33 mmol), Cs_2_CO_3_ (2.17 g, 6.7 mmol), compound 4b (2.23 g, 3.3 mmol), and L-proline (77 mg, 0.67 mmol) in DMF (50 mL). 2CzBBIT was obtained as a white solid (1.6 g, 85% yield). ^1^H NMR (500 MHz, CDCl_3_): δ (ppm) 8.30 (d, *J* = 1.8 Hz, 1H), 8.20-8.16 (m, 2H), 8.15-8.10 (m, 2H), 7.93-7.88 (m, 1H), 7.83-7.78 (m, 1H), 7.76 (dd, *J* = 8.5, 2.4 Hz, 1H), 7.69 (d, *J* = 2.1 Hz, 1H), 7.62 (d, *J* = 8.5 Hz, 1H), 7.57 (dd, *J* = 8.7, 2.0 Hz, 1H), 7.53-7.47 (m, 2H), 7.43-7.37 (m, 6H), 7.36-7.33 (m, 1H), 7.32-7.26 (m, 2H), 4.14 (s, 2H). ^13^C NMR (125 MHz, CDCl_3_): δ (ppm) 150.36, 144.02, 141.77, 141.44, 139.83, 134.81, 134.73, 132.58, 130.29, 127.67, 127.60, 126.89, 126.68, 125.87, 125.69, 124.55, 123.82, 123.38, 123.12, 123.07, 120.80, 120.75, 120.30, 119.80, 119.67, 119.62, 119.58, 111.35, 110.67, 109.92, 109.69, 30.31. MS (MALDI-TOF) [m/z]: Calcd for C_38_H_24_N_4_S, 568.172; Found, 568.152 [M^+^]. Elemental analysis (%) calcd for C_26_H_17_N_3_S: C 80.26, H 4.25, N 9.85, S 5.64. Found: C 80.17, H 4.36, N 9.92, S 5.57.

### Characterization

All reagents and solvents were purchased from Aldrich, Alfa Aesar, and TCI Chemicals, and used as received unless otherwise mentioned. ^1^H and ^13^C nuclear magnetic resonance spectra were recorded using Varian Mercury 500 MHz spectrometers in CDCl_3_ solvent (Cambridge Isotope Laboratories, Inc.) using tetramethylsilane as the internal standard. The elemental analysis was conducted using the EA1112 elemental analyzer (Thermo Electron Corporation) at the Center for Organic Reactions. The Bruker Daltonics LRF-20 MALDI-TOF spectrometer was utilized to record the mass spectrometry. The glass transition temperature (*T*_g_) was estimated by acquiring differential scanning calorimetry (DSC) thermograms measured using a Mettler 821 instrument under the nitrogen atmosphere at a heating rate of 10°C min^−1^. The decomposition temperature (*T*_d_) was obtained by conducting thermogravimetric analysis (TGA) using Mettler STAR^e^ at a heating rate of 10°C min^−1^ under the nitrogen atmosphere. The absorption spectra were attained using the Agilent 8453 UV–Vis (ultraviolet–visible) spectrophotometer. A Hitachi F-7000 fluorescence spectrophotometer was utilized to measure the photoluminescence (PL) spectra at RT. The HITACHI F-7000 fluorescence spectrometer was used to measure the phosphorescence spectra at a temperature of 77 K in the 2-methyltetrahydrofuran solvent. The JASCO FP-8500 fluorescence spectrometer equipped with an integrated sphere was utilized to measure the PL quantum yield (PLQY) for the blend films. CzBBIT: IAcTr-out (45 wt%) and 2CzBBIT: IAcTr-out (35 wt%) blend films were prepared with a thickness of 40 nm on a quartz substrate using the toluene solution to measure the PLQY. The same substrates were utilized to study the transient PL (TRPL) at RT. The PL decays were obtained between 0 and 50 μs. The TRPL and exciton lifetimes were restricted with a train of 1064 nm pulses at a 5 ns interval, produced at 10 Hz using an Nd:YAG laser (Powerlite Precision II 8000, Continuum). A wavelength was attained at 355 nm pulse by the generation of the third harmonic and used to directly excite the film sample prepared on quartz. The emission from the film sample was focused using a lens (focal length: 10 cm), passed through a monochromator, and distinguished by a photomultiplier tube connected to a 100 MHz digital oscilloscope (DSO-X 3014A, Keysight). The prompt and delayed components were recognized by single- and triple-exponential decaying functions. Cyclic voltammetry measurements were performed to evaluate the oxidation potential of host materials in their film state by employing a potentiostat (EA161, eDAQ) at a scan rate of 100 mV s^−1^. CzBBIT and 2CzBBIT dissolved in MC were drop-casted on a platinum (working) electrode and freshly prepared 0.1 M NBu_4_PF_6_ in dry acetonitrile was used as the electrolytic solution. Ag/AgCl was used as the reference electrode, and a platinum wire with a diameter of 0.5 mm was used as the counter electrode.

### Single-Carrier Devices

Single-carrier devices, such as hole-only devices (HODs) and electron-only devices (EODs), were fabricated using a neat glass substrate coated with indium tin oxide (ITO; thickness of 150 nm) with an active pattern size of 2 × 2 mm^2^ and a sheet resistance of 10 Ω cm^−2^, for use as the anode. The HODs were fabricated with the structure ITO (150 nm)/PEDOT:PSS (40 nm)/PVK (10 nm)/Host (30 nm)/Al (100 nm), and the EODs were fabricated with the structure ITO (150 nm)/Host (30 nm)/TPBi (40 nm)/LiF (0.8 nm)/Al (100 nm). PEDOT:PSS as a hole injection layer was deposited as received (annealed at 155°C for 15 min), PVK was dissolved in chlorobenzene (annealed at 130°C for 20 min), and the toluene solution of the host materials was spin-coated as required. Finally, the other essential layers, such as TPBi, LiF, and Al, were deposited using a vacuum thermal evaporator in a glove box.

### OLED Device Fabrication

The ITO-coated (150 nm) glass substrate with a sheet resistance of 10 Ω cm^−2^ and active pattern size of 2 × 2 mm^2^ acting as the anode was used to fabricate OLEDs. The IAcTr-out, which was reported by our group, was adopted as the green TADF emitter. The device structure was as follows: ITO (150 nm)/PEDOT:PSS (40 nm)/PVK (10 nm)/CzBBIT or 2CzBBIT: IAcTr-out (40 nm, x wt%)/TPBi (40 nm)/LiF (0.8 nm)/Al (100 nm), where x is the dopant concentration of 45.0 wt% for CzBBIT and 35.0 wt% for 2CzBBIT. The hole-injecting PEDOT:PSS was directly spin-coated on ITO and thermally annealed at 155°C for 15 min, whereas the hole-transporting PVK dissolved in chlorobenzene was spin-coated on PEDOT:PSS before annealing at 130°C for 20 min. The emissive layer was spin-coated on the PVK layer from the toluene solution. The aforementioned steps were conducted at ambient conditions, and the substrates were moved into a vacuum chamber for depositing TPBi, LiF, and Al sequentially using a thermal evaporator. The current density–voltage–luminance (*J*–*V*–*L*) data were measured using the Keithley SMU 236 instrument and SpectraScan PR-655 colorimeter. All the measurements were performed at atmospheric conditions without protecting the devices from any encapsulations.

## Results and Discussion

### Synthesis

**Scheme 1:**
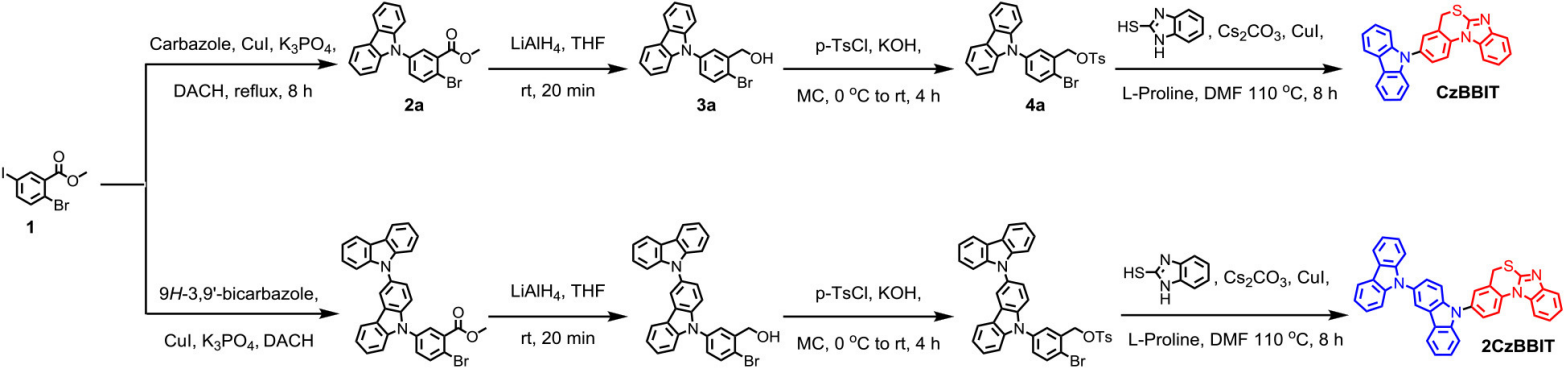
Synthetic route of CzBBIT and 2CzBBIT.

The synthetic details of both the targeted BBIT derivatives are shown in Scheme 1. Compound 1 was synthesized by adopting the reported synthetic procedure (Dressler et al., 2015). It was treated with 9*H*-carbazole or 9*H*-3,9′-bicarbazole, probing Cu(I)-catalyzed Ullmann reaction conditions using K_3_PO_4_ as the base and (±)-*trans*-1,2-diaminocyclohexane as the ligand to obtain 2a and 2b with moderate yields. Then, their ester groups were converted to the corresponding alcohols using LiAlH_4_ as the reducing agent with a reaction time of no longer than 20 min (Aline et al., 2008). Subsequently, the hydroxyl group was tosylated using p-toluenesulfonyl chloride and KOH as the base. Next, compounds 4a and 4b were treated with 2-mercaptobenzimidazole to provide CzBBIT and 2CzBBIT, respectively in high yields (refer to the experimental section for detailed information).

### Theoretical Calculations

**Figure 1:**
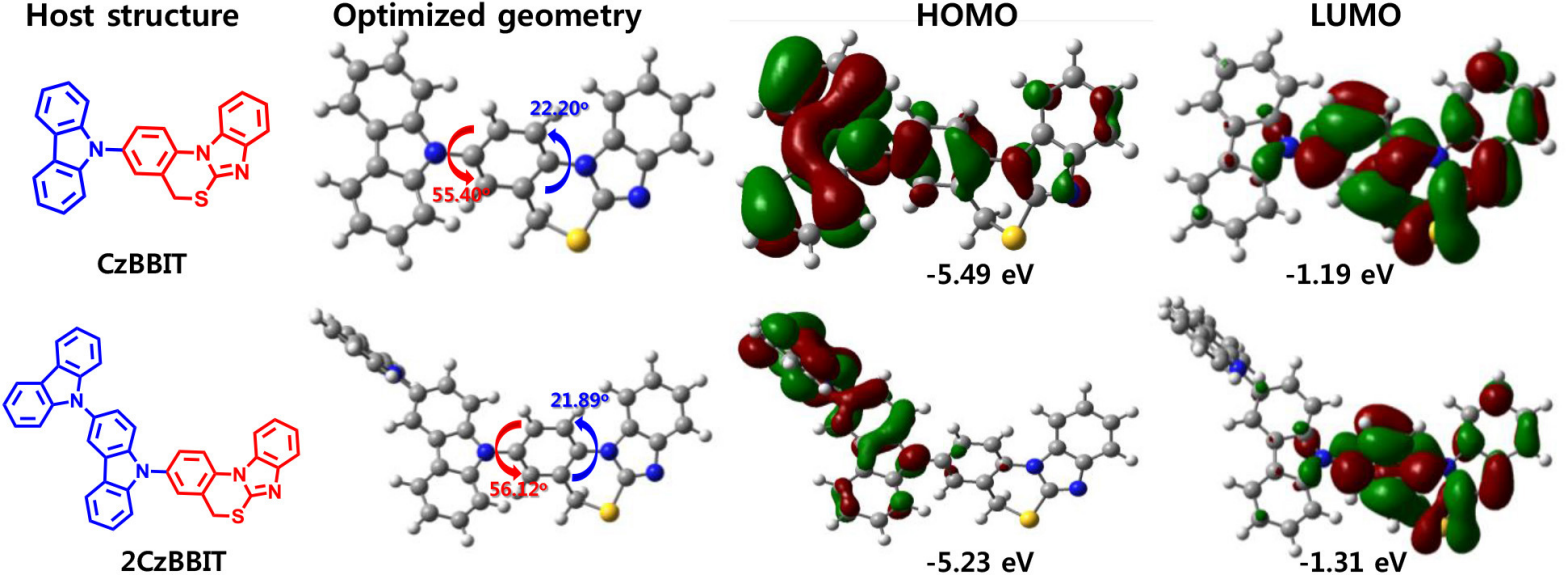
Chemical structure, optimized geometry, and highest occupied molecular orbital (HOMO) and lowest unoccupied molecular orbital (LUMO) spatial distributions of CzBBIT and 2CzBBIT calculated at the B3LYP/6-31G (DFT) level of theory with Gaussian 09W.

To obtain a deeper understanding of the molecular energy levels and energy distribution of CzBBIT and 2CzBBIT, density functional theory (DFT) and time-dependent DFT (TD-DFT) calculations using the B3LYP/6-31G(d) method were probed. The molecular structure, optimized geometry, and HOMO and LUMO spatial distributions of CzBBIT and 2CzBBIT calculated at the B3LYP/6-31G (DFT) level of theory with Gaussian 09W are presented in Figure 1. The dihedral angle perceived between the donor and BBIT entities of both hosts is approximately 56.0°, implying twisted geometry. BBIT itself possesses a dihedral angle of 22.0°, denoting that it does not demonstrate a planar structure. The electron cloud distributions of the HOMO are primarily localized on the electron-rich carbazole entities, which contribute to form a hole-transport channel. The LUMO is predominantly distributed over the electron-deficient BBIT core that provides the electron-transport channel. The well-separated HOMO and LUMO distributions could support the bipolar nature of the host materials. The HOMO/LUMO energy levels for CzBBIT and 2CzBBIT are calculated to be -5.49/-1.19 eV and -5.23/-1.31 eV, respectively. Besides, the *E*_T_ of CzBBIT is 3.17 eV, and that of 2CzBBIT is 3.14 eV. Therefore, it is established that the high *E*_T_ values and well-separated HOMO and LUMO distributions guaranteed these materials to function as bipolar hosts in OLEDs.

### Optical and Photophysical Properties

**Figure 2:**
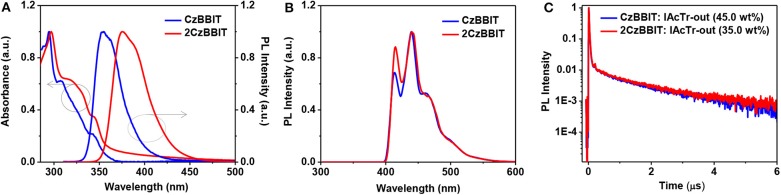
**(A)** UV–Vis absorption and photoluminescence (PL) spectra of CzBBIT and 2CzBBIT in toluene. **(B)** Phosphorescence spectra measured in 2-methyltetrahydrofuran at 77 K. **(C)** Transient PL decay curves of IAcTr-out doped films at room temperature.

The UV–Vis absorption and PL spectra were recorded in a toluene solution for CzBBIT and 2CzBBIT to evaluate their optical properties (Figure 2A and Table 1). Both compounds display similar absorption profiles with strong π-π^*^ transition bands under 300 nm, and the weak absorbance at about 330–350 nm mainly corresponds to the n-π^*^ transition of 9-phenyl carbazole units (Ma et al., 2019). The absorption profiles are not significantly influenced by the solution to film state (Figure S1 and Table 1), thereby revealing that there are negligible intermolecular interactions in the ground state. The optical bandgap (*E*_g_) values of CzBBIT and 2CzBBIT were derived from the absorption threshold in the film state as 3.44 and 3.32 eV, respectively.

**Table 1:** Photophysical and electrochemical data of hosts.

**Host**	***T***_**g**_/*T*_**d**_ **(°C)**	**Absorption (nm)**		**PL (nm)**		***E***_**g**_ **(eV)^**c**^**	***E***_**T**_ **(eV)^**d**^**	**Energy levels (eV)**	
		**Sol.**^**a**^	**Film**^**b**^	**Sol.**^**a**^	**Film**^**b**^			**HOMO**^**e**^	**LUMO**^**f**^
CzBBIT	106/333	294, 311, 341	297, 316, 344	355, 362	359, 367	3.44	3.0	−5.67	−2.23
2CzBBIT	161/392	295, 307, 343	297, 311, 345	376, 389	382, 394	3.32	3.0	−5.57	−2.25

a *Measured in toluene*.

b *Thin film*.

c *From the absorption threshold (film state)*.

d *Calculated from the phosphorescence spectra recorded in the 2-methyltetrahydrofuran*.

e *Calculated using oxidation onset in the film state*.

f *Calculated using HOMO + E_g_*.

As shown in Figure 2A, the PL spectrum of CzBBIT in toluene shows partially unstructured emissions with the 0-0 band at 355 nm and 0–1 band at 362 nm, respectively, while those two bands are observed at 376 nm and 388 nm for 2CzBBIT. The emission behavior at room temperature is due to charge-transfer excited states (Ma et al., 2019). The somewhat red-shifted emission for 2CzBBIT is attributed to the stronger electron-donating property for the bicarbazole donor than single carbazole.

Further, the absorption and emission spectra were not remarkably influenced by solvent polarity, indicating that the intramolecular charge-transfer nature is considerably weak in the ground and excited states (Figure S2 and Table S1). The phosphorescence spectrum was recorded at 77 K in 2-methyltetrahydrofuran for both compounds to obtain their *E*_T_, and the results are shown in Figure 2B. The spectra of CzBBIT and 2CzBBIT exhibit structured emission behavior with three vibronic peaks, which is mainly due to locally-excited (LE) states. The *E*_T_ values of both compounds are calculated from the highest energy vibronic sub-bands to be ~3.0 eV (Figure S3), which is sufficiently high for them to function as hosts for IAcTr-out, a TADF emitter (*E*_T_ of 2.62 eV) (Park et al., 2018). It should be noted that the high *E*_T_ observed for both host compounds could be expected from the interruption of π-conjugation by the non-conjugated methylene bridge.

### TRPL and Kinetic Parameters

TRPL studies were conducted to distinguish the TADF behavior of IAcTr-out doped into CzBBIT and 2CzBBIT in the film state under the nitrogen atmosphere (Figure 2C). It was also confirmed that both non-doped host films display only prompt (τ_p_) fluorescence lifetime, demonstrating typical fluorescence behavior. The τ_p_ values of CzBBIT and 2CzBBIT were obtained as 0.5 ns (Figure S4). On the other hand, both blend films display τ_p_ and delayed (τ_d_) fluorescence lifetimes to corroborate the presence of TADF behavior of IAcTr-out in both host environments. The τ_p_ and τ_d_ values were evaluated by fitting a double-exponential decay mode *I*(*t*) = *A*_1_ exp(−*t*/τ_p_) + *A*_2_ exp(−*t*/τ_d_), where *A*_1_ and *A*_2_ are fitting parameters (Xie et al., 2016). The τ_p_/τ_d_ values of CzBBIT and 2CzBBIT were obtained as 19.4 ns/1.94 μs and 20.9 ns/2.10 μs, respectively (Table S2). In addition, the absolute PLQY (Φ_PL_) values of the compounds were evaluated for the doped films using the integrating sphere. These values for CzBBIT and 2CzBBIT were observed to be 70.96 and 62.29%, respectively. However, the total Φ_PL_ of a TADF emitter comprises the prompt (Φ_p_) and delayed fluorescence (Φ_d_) components. The Φ_p_/Φ_d_ values of CzBBIT and 2CzBBIT were calculated to be 42.77%/28.19% and 37.84%/24.45%, respectively.

Besides, to obtain a deeper understanding of the TADF mechanism, we calculated the essential kinetic parameters, such as the singlet excited state radiative decay rate constant ($k_{r}^{s}$), intersystem crossing (ISC) rate constant from the singlet excited state to the triplet excited state (*k*_ISC_), RISC rate constant from the lowest triplet excited state to the lowest singlet excited state (*k*_RISC_), and non-radiative (nr) decay rate constant for the singlet excited state ($k_{nr}^{S}$), for the blended films (Table S2). Here, $k_{nr}^{T}$ at RT was intended to be zero (Tao et al., 2014). The *k*_RISC_ values of CzBBIT:IAcTr-out and 2CzBBIT:IAcTr-out were calculated to be 8.6 × 10^5^ s^−1^ and 7.9 × 10^5^ s^−1^, respectively. A faster *k*_RISC_ value resulted from the stabilized S_1_ state of the emitter in the host. The rate constants, such as prompt (*k*_p_ = 1/τ_p_) and delayed (*k*_d_ = 1/τ_d_) fluorescence decays, were deliberated using their corresponding prompt and delayed lifetimes.

### Electrochemical Properties

Cyclic voltammetry measurements were performed using a three-electrode electrochemical cell to evaluate the electrochemical properties of CzBBIT and 2CzBBIT in the film state (Figure S5 and Table 1). During the anodic sweep, both compounds exhibited a quasi-reversible oxidation peak instigated from the electron-rich carbazole/bicarbazole entities. The onset oxidation potentials ($E_{ox}^{onset}$) vs. Ag/Ag^+^ were determined as +1.23 and +1.13 V for CzBBIT and 2CzBBIT, respectively. The HOMO energy levels were estimated using the following equation: HOMO = -*e*(4.8 + $E_{ox}^{onset}$ - *E*_Fc/Fc+_) eV (Pommerehne et al., 1995), where the Fc/Fc^+^ potential was equal to +0.36 V. Accordingly, the HOMO levels were calculated as −5.67 and −5.57 eV, respectively, which are in line with DFT studies. The relatively shallower HOMO level attained for 2CzBBIT originated from the relatively stronger electron-donating ability of bicarbazole compared to the single carbazole derivative. The LUMO energy levels of CzBBIT and 2CzBBIT were determined to be −2.23 and −2.25 eV, respectively, by adding the *E*_g_ values to HOMO levels. The difference between the LUMO levels of both hosts was insignificant because of the identical electron-acceptor core.

### Thermal and Morphological Properties

The thermal stability of both new materials was examined using TGA and DSC measurements performed at a heating rate of 10°C min^−1^ under nitrogen (Figure S6 and Table 1). The TGA results corroborate that CzBBIT and 2CzBBIT have high decomposition temperatures (*T*_d_, corresponding to 5% weight loss) of 333°C and 392°C, respectively. The glass transition temperatures (*T*_g_) of CzBBIT and 2CzBBIT observed from the DSC thermograms are 106 and 161°C, respectively. The remarkable thermal stability of these two materials implies that both are potentially applicable in multilayered OLEDs fabricated through vacuum thermal deposition or solution processes. In particular, the enhanced thermal stability of 2CzBBIT compared to CzBBIT is due to the increased molecular weight. The thermal robustness of these materials is beneficial in producing smooth amorphous and homogeneous films during device fabrication, which is among the essential parameters to achieve a highly stable device performance.

The surface morphology of the blend films of CzBBIT: IAcTr-out and 2CzBBIT: IAcTr-out was examined by atomic force microscopy (Figure S7). Both the blend films are homogeneous, with very low root-mean-square roughness values of 0.30 nm for CzBBIT and 0.40 nm for 2CzBBIT. These results confirm that both hosts exhibit superior thermal and morphological stability, which is beneficial in enhancing device stability and electroluminescence (EL) performance during device operations.

### Single-Carrier Devices

The carrier-only devices, such as HODs and EODs, were fabricated, and their characteristics were examined to obtain a deeper insight into the carrier transport abilities of CzBBIT and 2CzBBIT. The device configuration of ITO (150 nm)/PEDOT:PSS (40 nm)/PVK (10 nm)/Host (30 nm)/Al (100 nm) was used for the HOD, and that of ITO (150 nm)/Host (30 nm)/TPBi (40 nm)/LiF (0.8 nm)/Al (100 nm) was employed for the EOD. The current density vs. voltage (*J*-*V*) curves of the corresponding devices are presented in Figure S8. Both hosts exhibit significant hole and electron current densities, which confirm their bipolar nature. However, both hosts exhibited that the electron current densities are much larger than the hole current densities at a given driving-voltage, demonstrating unbalanced charge transport. It can be assumed that the intrinsic electron character of BBIT is relatively high.

### Electroluminescence Properties

The excellent solubility, thermal robustness, tolerable electrochemical and optical properties, and bipolar nature of CzBBIT and 2CzBBIT propelled us to investigate them as host materials in solution-processable TADF-OLEDs. IAcTr-out was designated as the green TADF emitter because the triplet energy values of both hosts (~3.0 eV) are sufficiently higher than that of IAcTr-out (2.62 eV) (Park et al., 2018) to prevent back energy transfer from the dopant to host, in addition to other commensurate physical and electrochemical properties. Further, the absorption spectrum of IAcTr-out is well-overlapped with the emission spectrum of both hosts (Figure S9), which enable the efficient energy transfer from the host to the dopant. In particular, the PL spectrum of CzBBIT is more overlapped compared to that of 2CzBBIT and thus, a higher performance can be anticipated.

**Figure 3:**
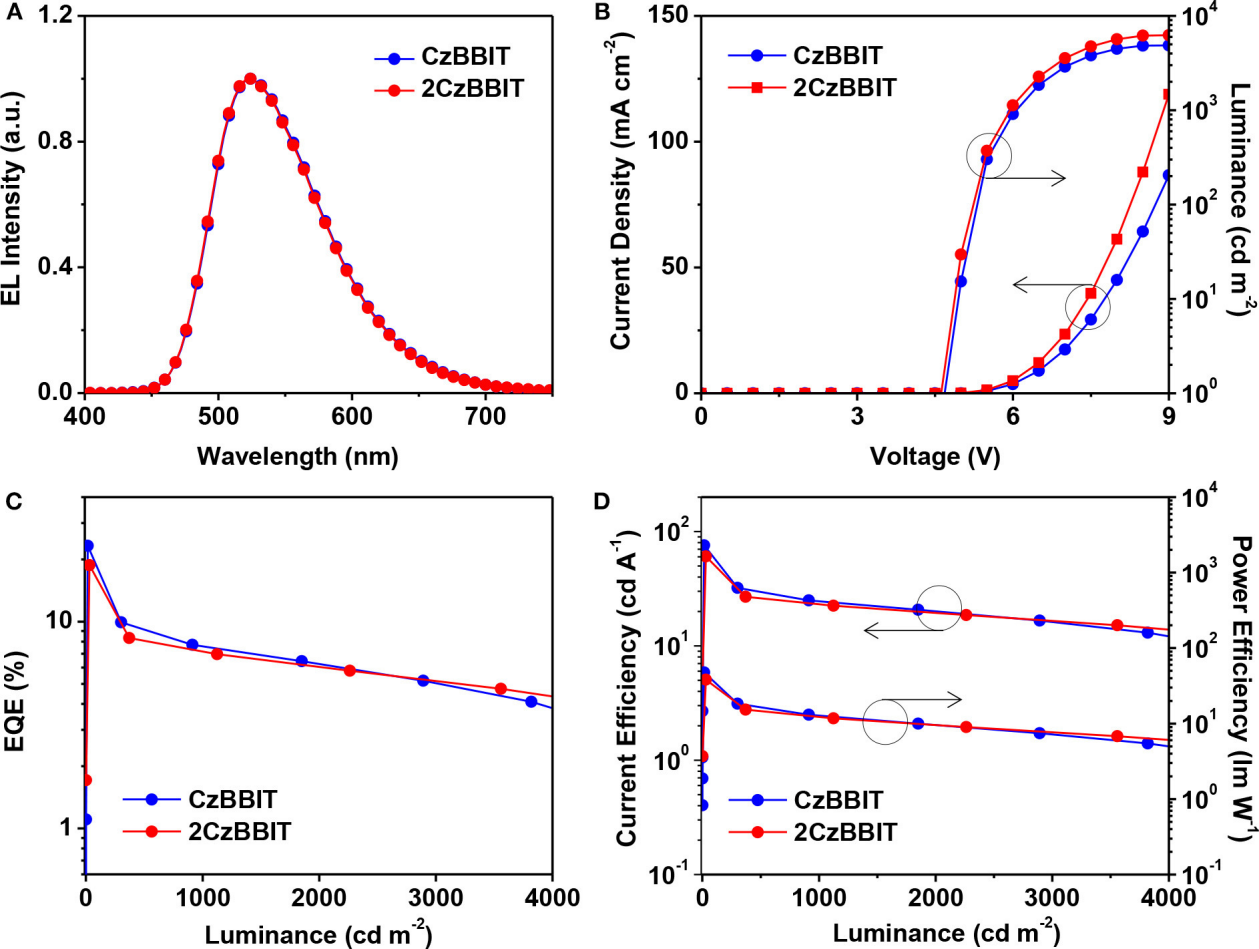
Characteristics of green thermally activated delayed fluorescence (TADF)-organic light-emitting diode (OLED) devices for CzBBIT: IAcTr-out and 2CzBBIT: IAcTr-out: **(A)** Normalized electroluminescence (EL) spectra (measured at 1,000 cd m^−2^). **(B)** Current density–voltage–luminance. **(C)** External quantum efficiency (EQE) vs. luminance. **(D)** Current efficiency and power efficiency vs. luminance plots.

The configuration of OLED was used as follows: ITO (150 nm)/PEDOT:PSS (40 nm)/PVK (10 nm)/CzBBIT or 2CzBBIT: IAcTr-out (40 nm, x wt%)/TPBi (40 nm)/LiF (0.8 nm)/Al (100 nm). PEDOT:PSS functioned as the hole-injection layer, PVK served as the hole-transporting layer, TPBi and LiF functioned as the electron-transport and electron-injection layers, respectively, and Al was the cathode. IAcTr-out doped into CzBBIT or 2CzBBIT was employed as the emissive layer with an optimized dopant concentration of 45.0 wt% for CzBBIT and 35.0 wt% for 2CzBBIT. The EL spectra, *J–V–L* spectra, EQE against luminance, and the current efficiency and power efficiency against the luminance curves are displayed in Figure 3. The pertinent data for the EL performance are summarized in Table 2. The schematic energy level diagram and chemical structures of the materials used in this study are shown in Figure S10.

**Table 2:** CzBBIT and 2CzBBIT based TADF-OLED performance data.

**Host**	***V*_**on**_ (V) ^**a**^**	**Quantum efficiency (%)**			**Current efficiency (cd A**^****−1****^**)**			**Power efficiency (lm W**^****−1****^**)**			**CIE color Coordinates (x, y)^**b**^**
		**Max**.	**100 cd/m^**2**^**	**500 cd/m^**2**^**	**Max**.	**100 cd/m^**2**^**	**500 cd/m^**2**^**	**Max**.	**100 cd/m^**2**^**	**500 cd/m^**2**^**	
CzBBIT	4.7	23.3	19.3	9.2	75.7	62.8	29.9	47.6	38.8	16.8	(0.33, 0.57)
2CzBBIT	4.6	18.7	16.7	8.1	60.6	53.7	14.8	38.1	40.4	14.8	(0.33, 0.57)

a *Turn-on voltage at a luminance of 1.0 cd m^−2^*.

b *At a luminance of 1,000 cd m^−2^*.

As shown in Figure 3A, the maximum EL wavelength of IAcTr-out in both host environments has appeared at a similar wavelength of 524 nm, with CIE color coordinates of (0.33, 0.57). The EL spectra obtained for both devices are similar to the PL spectra of the emitter in the film state (Figure S11). The absence of host emissions substantiates that complete energy transfer occurs from the host to the emitter, and all the electro-generated excitons are decayed through the emitter during radiative decay without any detrimental endothermic energy transfer. The similarity between the EL spectra in both host circumstances is attributed to the insignificant polarity variations, as evidenced by the solvatochromic PL studies.

Remarkable performances were achieved in the case of both the devices with an EQE/current efficiency/power efficiency of 23.3 %/75.7 cd A^−1^/47.6 lm W^−1^ for CzBBIT and 18.7%/60.6 cd A^−1^/38.1 lm W^−1^ for 2CzBBIT, respectively. The EQE was retained at 19.3 and 16.7% for CzBBIT and 2CzBBIT, respectively at a luminance of 100 cd m^−2^. However, the EQE in the devices of CzBBIT and 2CzBBIT decreased sharply to 9.2 and 8.1%, respectively, at a brightness of 500 cd m^−2^, indicating significant efficiency roll-off. This can be assumed to be due to unbalanced charge transport in the EML of both devices.

The EQEs of both the devices are much superior to the theoretical EQE limitation for conventional fluorescent emitters (~5%). This is practical evidence for IAcTr-out that utilizes triplet excitons generated after electric excitation through an efficient RISC process during the EL emission. The results verified that BBIT is an outstanding electron-acceptor core that can include a variety of bipolar host materials to achieve high performances in TADF-OLEDs.

## Conclusion

In conclusion, benzo[4,5]imidazo[2,1-b][1,3]thiazine (BBIT) as an electron-acceptor core was introduced for the first time and two new bipolar host materials (CzBBIT and 2CzBBIT) were developed by tethering electron-rich carbazole/bicarbazole entities as donors. The benzimidazole moiety provided an electron-deficient nature to enable an electron-transport nature and benzothiazine functioned as the backbone to BBIT. Besides, the non-conjugated sp^3^ hybridized methylene bridge was beneficial to retain the high *E*_T_ by interrupting electron delocalization and partially enabling the solubility. The systematic investigations proved that both hosts not only exhibited a high *E*_T_ value (3.0 eV) and a bipolar nature but also revealed thermal robustness and appropriate optical and electrochemical properties. As a result, the TADF-OLEDs fabricated through the solution process by employing CzBBIT and 2CzBBIT as hosts in the emissive layer demonstrated excellent performances with the maximum EQE values of 23.3 and 18.7%, respectively. The superior electron current density and state-of-the-art device performances substantiate that BBIT is an emerging electron-acceptor core to include versatile host materials suitable for OLEDs. As this is the first report about the BBIT core in OLED applications, there is much room to promote in the future.

## Data Availability Statement

The raw data supporting the conclusions of this article will be made available by the authors, without undue reservation, to any qualified researcher.

## Author Contributions

MG designed and synthesized the two new host materials, conducted the photophysical, electrochemical, device characterization, and drafted the entire manuscript. JY, J-EJ, and HW evaluated the thermal and PLQY measurements. SYP provided support during OLED fabrication. CL, YK, and SP conducted the TRPL measurements. MC advised on the synthesis. DC supervised the work and provided many suggestions in all aspects.

### Conflict of Interest

The authors declare that the research was conducted in the absence of any commercial or financial relationships that could be construed as a potential conflict of interest.

## Supplementary Material

The supplementary material includes the synthesis of key intermediates; UV-Vis absorption and PL spectra of CzBBIT and 2CzBBIT in film states and in different polar solvents; Fluorescence and phosphorescence spectra of CzBBIT and 2CzBBIT measured in 2-methyltetrahydrofuran; Cyclic voltammograms, thermogravimetric analysis and differential scanning calorimetry thermograms of CzBBIT and 2CzBBIT; Atomic force microscopy topographic images of blend films; Hole-only devices (HOD) and electron-only devices (EOD) for CzBBIT and 2CzBBIT; UV-Vis absorption spectra of the dopant IAcTr-out and PL spectra of CzBBIT and 2CzBBIT in the film state; Energy-level diagram and chemical structures of the materials used in this study.
