# Supplementary material for: 5H-Benzo[d]Benzo[4,5]Imidazo[2,1-b][1,3]Thiazine as a Novel Electron-Acceptor Cored High Triplet Energy Bipolar Host Material for Efficient Solution-Processable Thermally Activated Delayed Fluorescence Organic Light-Emitting Diodes
Source: Front Chem. 2020 Feb 7;8:61. doi: 10.3389/fchem.2020.00061 (PMC7020745; doi:10.3389/fchem.2020.00061)
Supplement: Supplementary file 1 [file Data_Sheet_1.pdf]

Supplementary Material

5H-Benzo[d]benzo[4,5]imidazo[2,1-b][1,3]thiazine as a Novel Electron-Acceptor Cored High Triplet Energy Bipolar Host Material for Efficient Solution-Processable Thermally Activated Delayed Fluorescence-Organic Light-Emitting Diodes

Mallesham Godumala, Jiwon Yoon, Seo Yeon Park, Chiho Lee, Youngseo Kim, Ji-Eun Jeong, Sungnam Park, Han Young Woo, Min Ju Cho and Dong Hoon Choi*

Department of Chemistry, Research Institute for Natural Sciences, Korea University, 145, Anam-ro, Sungbuk-gu, Seoul 02841, Korea.

*Corresponding author: dhchoi8803@korea.ac.kr

Experimental

Synthesis of methyl 2-bromo-5-(9H-carbazol-9-yl)benzoate (2a):

9H-carbazole (2.0 g, 12.0 mmol), compound 1 (4.48 g, 13.2 mmol), K_3PO_4 (7.63 g, 36.0 mmol), CuI (0.46 g, 2.4 mmol), and (±)-*trans*-1,2-diaminocyclohexane (0.72 mL, 6.0 mmol) were mixed in dry toluene (60 mL) and evacuated with nitrogen for 20 min. The reaction mixture was stirred at a refluxing temperature for 8 h. It was then cooled to room temperature (RT), filtered, and washed with MC (150 mL). The accumulated organic layer was concentrated in vacuo and column-chromatographed using 5% EA in hexane eluent. The resulting solid was further purified by precipitating in methanol to obtain 2a as a white solid (1.9 g, 42% yield).). ^{1}H NMR (500 MHz, $CDCl_3$): δ (ppm) 8.13 (d, *J* = 7.9 Hz, 2H), 8.02 (d, *J* = 2.4 Hz, 1H), 7.89 (d, *J* = 8.5 Hz, 1H), 7.55 (dd, *J* = 8.5, 2.7 Hz, 1H), 7.42 (ddd, *J* = 8.2, 7.1, 0.9 Hz, 3H), 7.37 (d, *J* = 8.2 Hz, 3H), 7.33-7.28 (m, 2H), 3.94 (s, 3H). ^{13}C NMR (125 MHz, $CDCl_3$): δ (ppm) 165.76, 140.37, 137.09, 135.97, 133.68, 130.98, 129.78, 126.25, 123.63, 120.53, 120.48, 120.01, 109.41, 52.77.

Synthesis of methyl 5-(9H-[3,9'-bicarbazol]-9-yl)-2-bromobenzoate (2b):

The synthetic procedure described for compound 2a was adopted to prepare compound 2b by combining 9H-3,9'-bicarbazole (4.0 g, 12.0 mmol), compound 1 (4.50 g, 13.2 mmol), K_3PO_4 (7.67 g, 36.1 mmol), CuI (0.46 g, 2.4 mmol), and (±)-*trans*-1,2-diaminocyclohexane (0.72 mL, 6.0 mmol) in toluene, which resulted in a white solid (3.0 g, 46% yield). ^{1}H NMR (500 MHz, $CDCl_3$): δ (ppm) 8.29-8.27 (m, 1H), 8.20-8.16 (m, 2H), 8.12-8.10 (m, 1H), 7.95 (d, *J* = 8.5 Hz, 1H), 7.64 (dd, *J* = 8.2, 2.6 Hz, 1H), 7.56 (dd, *J* = 2.1, 1.2 Hz, 2H), 7.50-7.48 (m, 1H), 7.45-7.37 (m, 6H), 7.36-7.27 (m, 3H), 3.98 (s, 3H). ^{13}C NMR (125 MHz, $CDCl_3$): δ (ppm) 165.92, 141.98, 141.32, 136.99, 136.40, 131.27, 131.21, 130.71, 130.04, 127.21, 126.10, 126.02, 124.92, 123.42, 123.40, 123.36, 121.19, 120.98, 120.73, 120.52, 119.91, 119.87, 110.76, 110.00, 109.93, 53.08.

Synthesis of (2-bromo-5-(9H-carbazol-9-yl)phenyl)methanol (3a):

To the solution of $LiAlH_4$ (0.22 g, 6.3 mmol) in THF (10 mL) at 0 ℃, the compound 2a (2.0 g, 5.3 mmol) dissolved in THF (15 mL) was gradually added, and the reaction mixture was vigorously stirred for 20 min at the same temperature. The remaining metal hydride was carefully quenched with water, and the organic compound was extracted with EA. The combined organic layers were dried and concentrated, and the residue was purified by silica gel column chromatography with 1:4 ratios of hexane and ethyl acetate as the eluent to yield 3a as a white solid (1.7 g, 91.8% yield). ^{1}H NMR (500 MHz, $CDCl_3$): δ (ppm) 8.13 (dt, *J* = 7.9, 1.0 Hz, 2H), 7.75 (d, *J* = 8.2 Hz, 1H), 7.73 (d, *J* = 2.4 Hz, 1H), 7.43-7.35 (m, 5H), 7.31-7.26 (m, 2H), 4.84 (d, *J* = 6.1 Hz, 2H), 2.05 (t, *J* = 6.1 Hz, 1H). ^{13}C NMR (125 MHz, $CDCl_3$): δ (ppm) 141.77, 140.59, 137.40, 133.89, 127.36, 127.01, 126.07, 123.48, 120.38, 120.35, 120.20, 109.62, 64.72.

Synthesis of (5-(9H-[3,9'-bicarbazol]-9-yl)-2-bromophenyl)methanol (3b):

Following the procedure used for compound 3a, compound 2b (2.0 g, 3.7 mmol) and $LiAlH_4$ (0.15 g, 4.4 mmol) were reacted in THF to produce 3b as a white solid (1.6 g, 84.3% yield). ^{1}H NMR (500 MHz, $CDCl_3$): δ (ppm) 8.29-8.26 (m, 1H), 8.18 (d, *J* = 7.6 Hz, 2H), 8.10 (d, *J* = 7.6 Hz, 1H), 7.84-7.79 (m, 2H), 7.59-7.52 (m, 2H), 7.50-7.37 (m, 7H), 7.34-7.27 (m, 3H), 4.88 (d, *J* = 6.1 Hz, 2H), 2.12 (t, *J* = 6.1 Hz, 1H). ^{13}C NMR (125 MHz, $CDCl_3$): δ (ppm) 142.08, 141.84, 137.14, 134.09, 130.18,127.40, 127.11, 127.03, 126.83, 125.87, 125.67, 123.31, 123.13, 123.07, 120.67, 120.30, 120.28, 119.66, 119.64, 119.58, 110.78, 110.76, 110.02, 109.78, 109.22, 64.70.

Synthesis of 2-bromo-5-(9H-carbazol-9-yl)benzyl 4-methylbenzenesulfonate (4a):

p-toluenesulfonyl chloride (1.17 g, 6.2 mmol) in MC (10 mL) was added to a stirred solution of compound 3a (1.8 g, 5.1 mmol) and KOH (2.3 g, 41.0 mmol) in MC (15 mL) at 0 ℃. After stirring at the same temperature for 20 min, the mixture was allowed to cool to RT, and stirring was continued for another 4 h. The excess KOH was quenched with water (50 mL), and the organic compound was extracted with MC (3 X 30 mL). The organic extract was dried over Na_2SO_4, concentrated, and purified through column chromatography using hexane: MC (1:2) as the eluent to produce 4a as a colorless solid (2.2 g, 85% yield). ^{1}H NMR (500 MHz, $CDCl_3$): δ (ppm) 8.13 (d, *J* = 7.6 Hz, 2H), 7.81 (d, *J* = 8.2 Hz, 2H), 7.73 (d, *J* = 8.5 Hz, 1H), 7.54 (d, *J* = 2.4 Hz, 1H), 7.44-7.38 (m, 3H), 7.33-7.29 (m, 4H), 7.29-7.27 (m, 2H), 5.23 (s, 2H), 2.35 (s, 3H).

Synthesis of 5-(9H-[3,9'-bicarbazol]-9-yl)-2-bromobenzyl 4-methylbenzenesulfonate (4b)

Following the procedure used for compound 4a, compound 3b (2.2 g, 4.3 mmol), *p*-toluenesulfonyl chloride (0.98 g, 5.1 mmol), and KOH (1.91 g, 34.1 mmol) were reacted in MC to produce 4b as a colorless solid (2.5 g, 88.0% yield). ^{1}H NMR (500 MHz, $CDCl_3$): δ (ppm) 8.28 (d, J = 1.5 Hz, 1H), 8.19 (d, J = 7.9 Hz, 2H), 8.11 (d, J = 7.6 Hz, 1H), 7.87-7.84 (m, 2H), 7.82 (d, J = 8.2 Hz, 1H), 7.68 (d, J = 2.4 Hz, 1H), 7.58-7.54 (m, 1H), 7.53-7.29 (m, 13H). ^{13}C NMR (125 MHz, $CDCl_3$): δ (ppm) 145.25, 141.82, 139.53, 137.18, 135.48, 134.49, 130.39, 129.98, 128.71, 128.26, 128.08, 128.06, 126.94, 125.90, 125.75, 124.65, 123.16, 123.13, 121.49, 120.88, 120.74, 120.31, 119.69, 119.64, 110.63, 109.86, 109.77, 70.28, 31.59.

Time-Resolved Photoluminescence of CzBBIT and 2CzBBIT

Time-resolved PL signals, $S(t)$, of the non-doped films of CzBBIT and 2CzBBIT were collected by using a time-correlated single-photon counting (TCSPC) technique. The films were excited by a 375 nm pulse (LDH-P-C-520, PicoQuant). The fluorescence emission of the films was measured at 420 nm by using a photomultiplier tube (PMA 182, PicoQuant). The TRPL decay curve was fitted by a double-exponential function, $S(t) = \Sigma^{2}_{i=1} A_i \exp(-t/\tau_i)$.

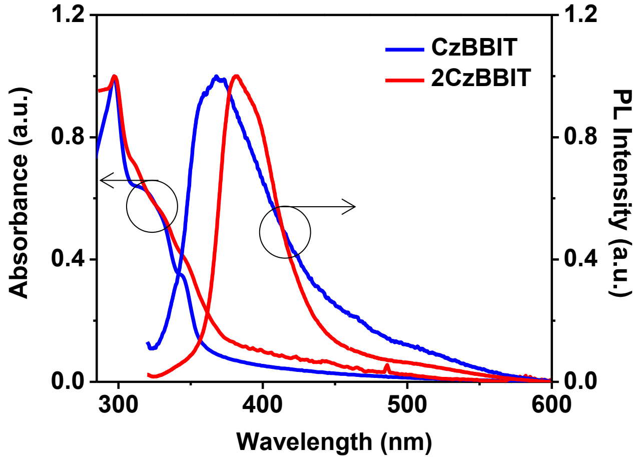

Figure S1. UV–Vis absorption and PL spectra of CzBBIT and 2CzBBIT in film states.

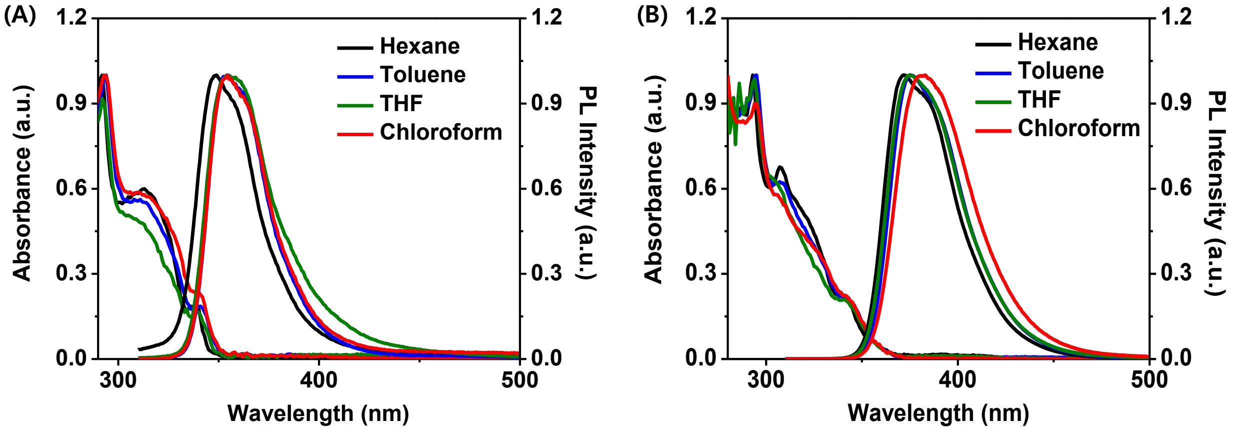

Figure S2. UV–Vis absorption and PL spectra of **(A)** CzBBIT and **(B)** 2CzBBIT in different polar solvents.

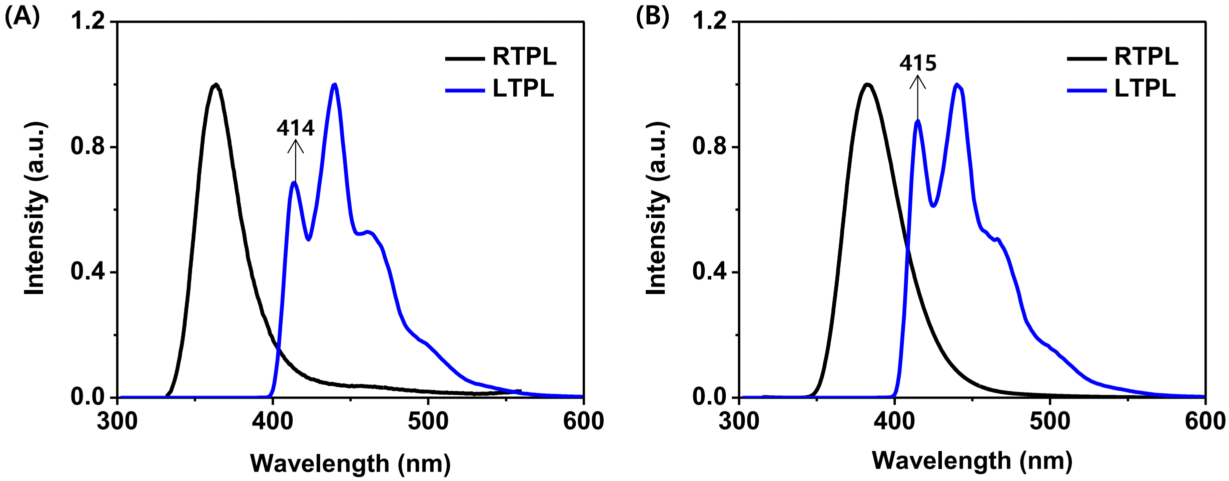

Figure S3. Fluorescence and phosphorescence spectra of **(A)** CzBBIT and **(B)** 2CzBBIT measured in 2-methyltetrahydrofuran.

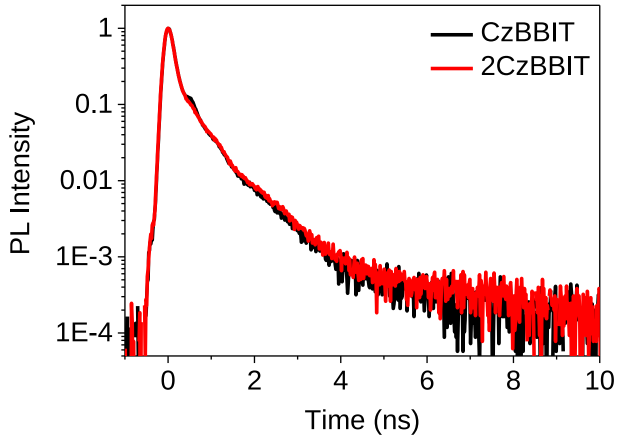

Figure S4. Time-resolved photoluminescence spectroscopy of the CzBBIT and 2CzBBIT films at room temperature.

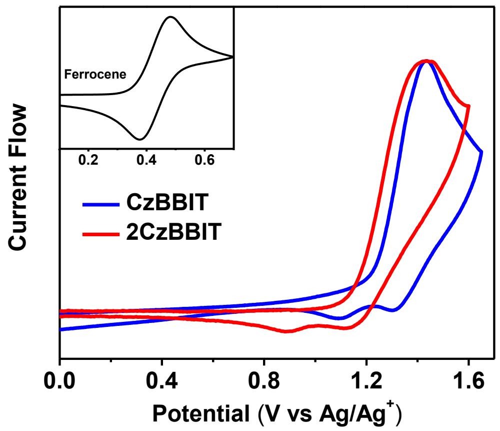

Figure S5. Cyclic voltammograms of CzBBIT and 2CzBBIT in film states (the cyclic voltammogram of ferrocene is shown as insert figure).

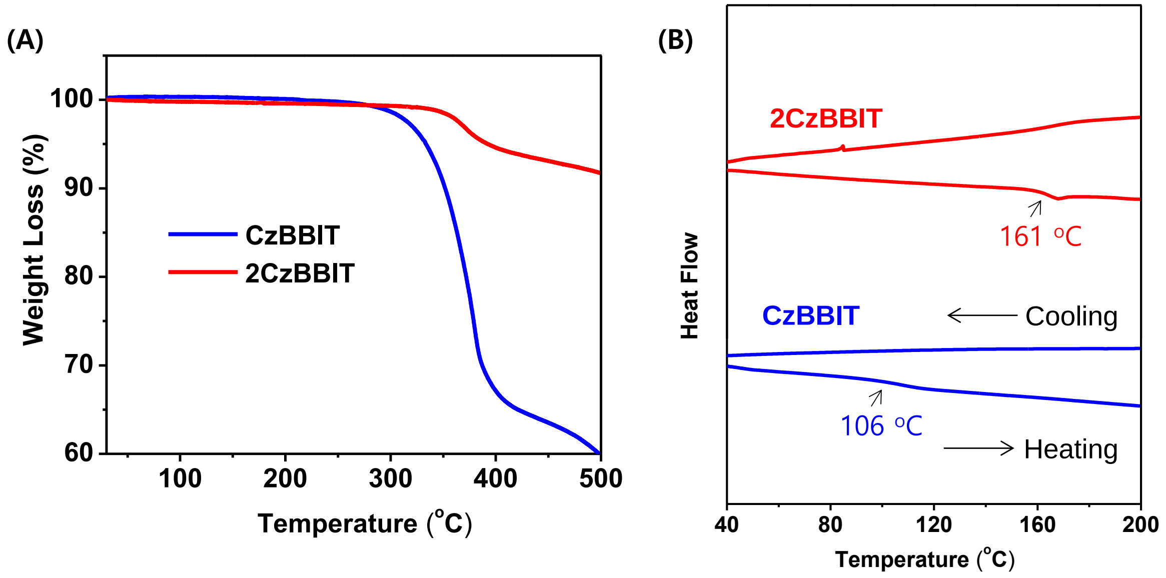

Figure S6. **(A)** Thermogravimetric analysis and **(B)** differential scanning calorimetry thermograms of CzBBIT and 2CzBBIT.

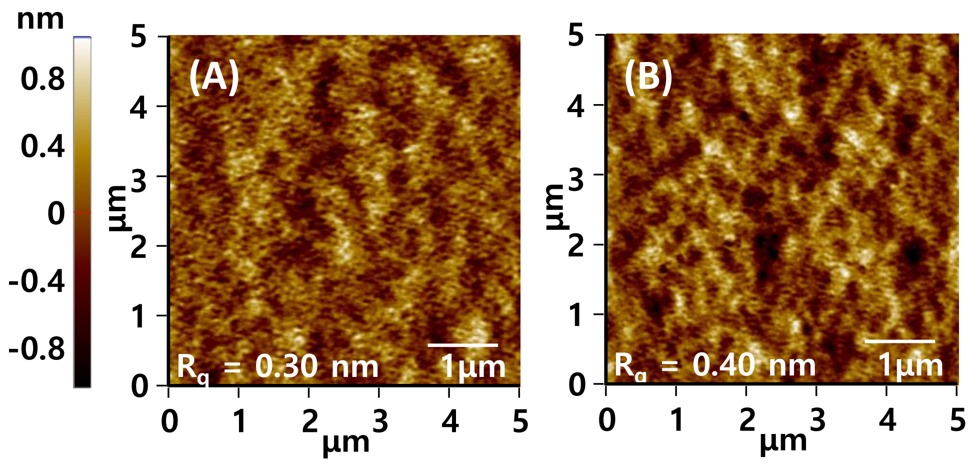

Figure S7. Atomic force microscopy topographic images (5 × 5 μm) of blend films of **(A)** CzBBIT: IAcTr-out and **(B)** 2CzBBIT: IAcTr-out.

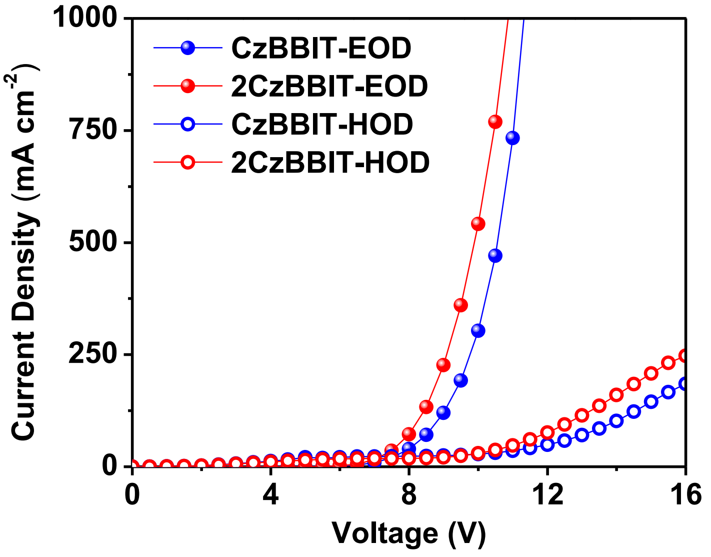

Figure S8. Hole-only devices (HOD) and electron-only devices (EOD) for CzBBIT and 2CzBBIT.

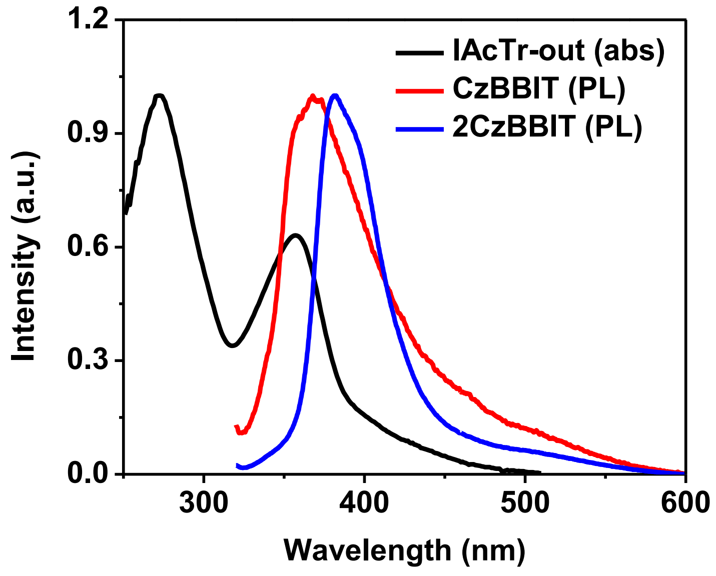

Figure S9. UV–Vis absorption spectra of the dopant IAcTr-out and PL spectra of CzBBIT and 2CzBBIT in film states.

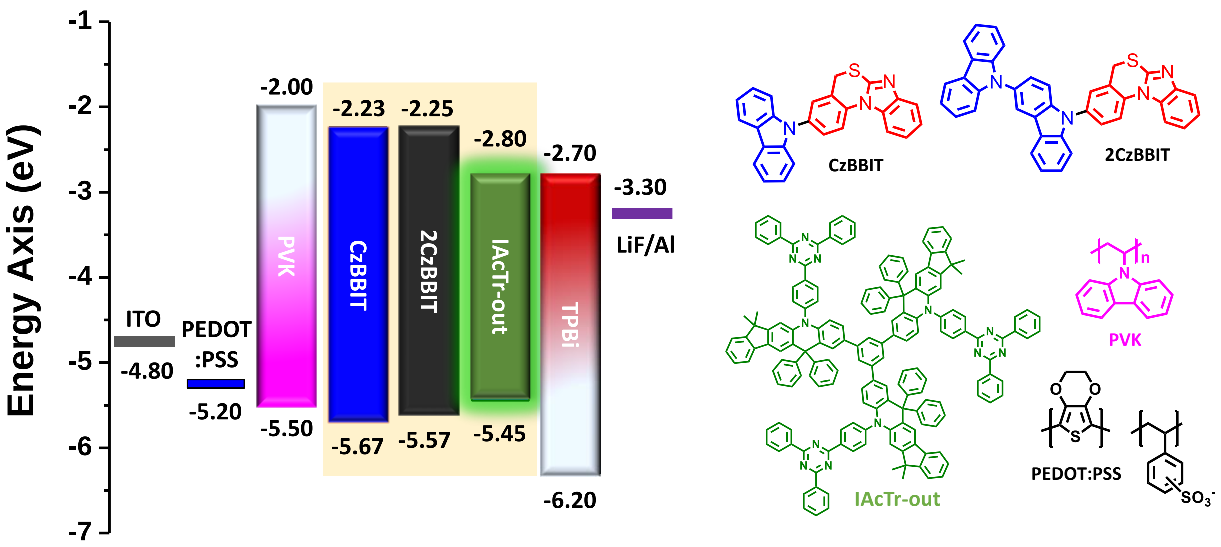

Figure S10. Energy-level diagram and chemical structures of the materials used in this study.

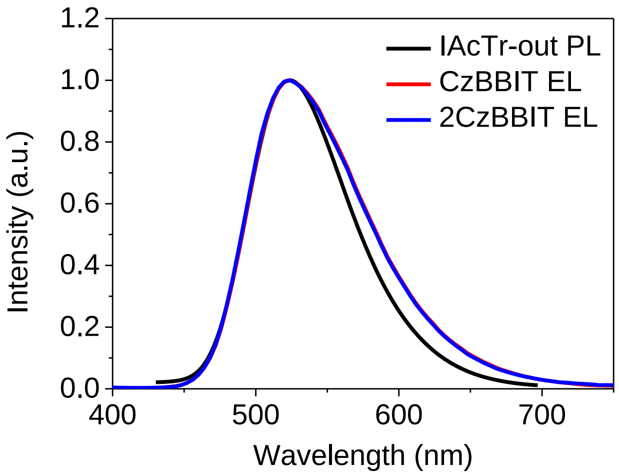

Figure S11. Normalized PL spectrum of the dopant IAcTr-out in film state and normalized EL spectra (measured at 1000 cd m^{-2}) of CzBBIT:IAcTr-out and 2CzBBIT:IAcTr-out based devices.

Table S1. UV-Vis absorption and PL spectroscopic data of two hosts in different polar solvents.

**Host**	Hexane [a/b] (nm)	Toluene [a/b] (nm)	THF [a/b] (nm)	Chloroform [a/b] (nm)
CzBBIT	292, 313/349	294, 308/355	285, 313/354	293, 313/354
2CzBBIT	293, 307, 339/372	295, 307, 339/376	294, 307, 341/375	295, 305, 340/383

[a] Absorption bands. [b] PL emission band

Table S2. TRPL and kinetic properties of the blend films of CzBBIT: IAcTr-out and 2CzBBIT: IAcTr-out.

**Host**	τ_p (ns)	τ_d (µs)	Φ_{PL} (%)	Φ_p (%)	Φ_d (%)	k_p (x 10^7 s^{-1})	k_d (x 10^5 s^{-1})	k_r^s (x 10^7 s^{-1})	k_{ISC} (x 10^7 s^{-1})	k_{RISC} (x 10^5 s^{-1})	k_{nr}^s (x 10^6 s^{-1})
**CzBBIT**	19.4	1.94	70.96	42.77	28.19	5.2	5.2	2.20	2.05	8.6	9.0
**2CzBBIT**	20.9	2.10	62.29	37.84	24.45	4.8	4.8	1.81	1.88	7.9	11.0

τ_p is the prompt emission lifetime, τ_d is the delayed emission lifetime, Φ_{PL} is the total PLQY, Φ_P is the prompt PLQY, Φ_d is the delayed PLQY, k_p is the prompt emission rate constant, k_d is the delayed emission rate constant, k_r^s is the radiative decay rate constant of the singlet excited state, k_{ISC} is the intersystem crossing rate constant, k_{RISC} is the reverse intersystem crossing rate constant, and k_{nr}^s is the non-radiative decay rate constant for singlet excited state.
